# Sitting and caregiver speech input in typically developing infants and infants with cerebral palsy

**DOI:** 10.1371/journal.pone.0324106

**Published:** 2025-05-23

**Authors:** Kari S. Kretch, Emily C. Marcinowski, Natalie A. Koziol, Regina T. Harbourne, Lin-Ya Hsu, Michele A. Lobo, Sandra L. Willett, Stacey C. Dusing

**Affiliations:** 1 Division of Biokinesiology and Physical Therapy, University of Southern California, Los Angeles, California, United States of America; 2 Department of Kinesiology, Louisiana State University, Baton Rouge, Louisiana, United States of America; 3 Nebraska Center for Research on Children, Youth, Families, and Schools, University of Nebraska-Lincoln, Lincoln, Nebraska, United States of America; 4 John G. Rangos School of Health Sciences, Duquesne University, Pittsburgh, Pennsylvania, United States of America; 5 Department of Rehabilitation Medicine, University of Washington, Seattle, Washington, United States of America; 6 Department of Physical Therapy, University of Delaware, Newark, Delaware, United States of America; 7 Department of Kinesiology, Colorado Mesa University, Colorado, United States of America; Southwest University, CHINA

## Abstract

The development of independent sitting is associated with language development, but the learning experiences underlying this relationship are not well understood. Additionally, it is unknown how these processes play out in infants with motor impairments and delays in sitting development. We examined the real-time associations between sitting and caregiver speech input in 28 5–7-month-old infants with typical development and 22 7–16-month-old infants with cerebral palsy who were at a similar stage of early sitting development. We hypothesized that object labels would be more likely to co-occur with moments of optimal attention to the labeled object while sitting than while in other positions. Infants were video recorded in five minutes of free play with a caregiver. Coders transcribed caregivers’ speech, identified instances of object labeling, and coded infants’ and caregivers’ attentional states during object labeling episodes. We found that caregivers labeled more objects while infants were sitting than while they were in other positions. However, object labels were not more likely to co-occur with infant attention, infant multimodal attention, or coordinated visual attention to the labeled object during sitting. Infants with cerebral palsy were exposed to fewer labels and were less likely to be attending to objects as they were labeled than infants with typical development. Our findings shed light on a possible pathway connecting sitting and language in typical and atypical development.

## Introduction

The most salient developments of infancy are the emergence of motor and language skills. In the first two years of life, typically developing infants learn to coordinate small and large body movements to sit upright, walk, and manipulate objects, and to engage in functional verbal communication with social partners. A wealth of recent empirical and theoretical work has examined how these two domains are linked during this formative developmental period [1]. The bulk of this research focuses on relations between walking and language development [2–10], as these skills emerge on a similar time frame, with the onset of independent walking and production of first words both occurring around 12 months of age.

However, even earlier developments in motor skill have been linked to language outcomes. In particular, several studies have found associations between the development of sitting and both receptive and productive language measures [5,6,11]. This is surprising because sitting typically develops around 6 months of age, long before infants utter their first words. However, infants demonstrate understanding of many common nouns as early as 6 months [12,13], suggesting that the seeds for language learning are planted early.

The most immediate effect of improvement in sitting skill is infants spending more time in a sitting position during play and throughout their everyday activities [14,15]. How might being in a sitting position relate to language development? Possibly, sitting enables or increases specific sensorimotor interactions that are beneficial for word learning. Thus, it is crucial to examine how sitting impacts the language learning environment in *real time*—the immediate time scale of behaviors and events. Understanding the real-time behaviors that co-occur with sitting can provide clues about how sitting may impact development [16–18]. Notably, most of the first words infants produce and understand are concrete nouns [12,13,19–21]; therefore, characterizing the everyday interactions in which young infants are exposed to the names of objects is of particular interest.

In the second year of life, object labeling is tightly linked to infants’ ongoing actions. Caregivers tend to label or describe objects that infants are touching, holding, and actively manipulating, and objects at which infants are looking [22–25]. Holding objects brings them close to infants’ faces, making them visually dominant in the infant’s field of view [26,27], and helps to clarify which of the many objects in the environment the caregiver is labeling [28]. Less is known about the context of object labeling at earlier points in development; however, as early as 3 months of age, object naming appears linked to infants’ active engagement with objects [29,30].

This elegant coordination between visual, manual, and verbal input, referred to aptly by West and Iverson [24] as “the right label at the right time,” is advantageous for language learning. In laboratory studies, novel object names that are uttered while being held by the infant, and therefore visually dominant, are more likely to be learned than names that are spoken in less optimal contexts [26]. In particular, the multimodal combination of simultaneous infant looking and touching during labeling has the strongest associations with word learning [31]. On a longer timescale, infants’ vocabularies are biased toward names of items they manipulate during their everyday interactions [19]. Referential transparency—how well a naïve observer can guess the word a caregiver is uttering in a muted video using visual cues like looking and touching [28]—in interactions at 14 and 18 months of age predicts children’s vocabulary at 4.5 years [32]. Similarly, infants whose parents spent more time talking about objects the child was attending to at 9 months of age had better language skills at 12 and 30 months of age [33]. Increased rates of co-occurrence between caregiver object labels and infant looking and/or touching may underlie the impact of constructs known to be linked to language outcomes, including maternal sensitivity or responsiveness [34,35] and joint attention [36,37].

How might infants’ emerging sitting skills contribute to the language learning environment? Sitting provides an optimal stable position for visual and manual exploration of objects [17,38,39], potentially providing more opportunities for coordinated parent labeling. The upright head position facilitates visual access to the surroundings [40], making it easier for infants and caregivers to monitor the focus of the others’ attention [16]. Independent sitting also affects the physical configuration of infants and caregivers during object play, with infants who can sit independently less likely to be facing away from their caregivers [39] and more likely to sit at right angles [41], providing an ideal physical context for social and object interaction [18]. Therefore, we hypothesize that sitting may increase the co-occurrence of object labels and infant visual and manual attention. However, this hypothesis has yet to be examined empirically.

The role of sitting in typical language development implies that delays in sitting development may have downstream effects on language development in infants with motor impairments [42]. Cerebral palsy—the most common motor disability in childhood [43]—is by definition a disorder of posture and movement [44]. However, cerebral palsy is also associated with poor communication outcomes: It is estimated that 50–85% of individuals with cerebral palsy have deficits in speech and language [45–49]. Contributors to language impairments include oral-motor difficulties [50] and intellectual disability [51,52]. Interestingly, language and communication outcomes in children with cerebral palsy are associated with gross motor function [52–54]. Potentially, delays in early motor skills and effects on caregiver-infant interactions may be a compounding factor.

Some studies suggest that the facilitative effects of sitting on play and interaction in infants with typical development, as reviewed above, are also seen in infants with motor impairments. For example, caregivers provide overall richer cognitive learning opportunities when infants are in a sitting position, and this effect is similar in typically developing infants and infants with gross motor delay [18]. Similarly, infants who require caregiver support for sitting—including both 5–7-month-old typically developing infants and 7–16-month-old infants with motor delay—spend more time facing away from their caregivers and less time interacting with objects than their peers who sit independently [39]. Developmentally, improvements in object manipulation follow the onset of independent sitting both in infants with typical development and in those with delayed sitting development [55,56]. Therefore, delays in sitting may impede caregivers’ ability to provide object labels that co-occur with infant attention. However, links between sitting and speech input in infants with typical and atypical trajectories of sitting development have not yet been described. Examining impacts of sitting on caregiver speech input in both groups of infants can assess the universality of the hypothesized developmental phenomenon, and elucidate whether developmental cascades initiated by early motor skills are disrupted or merely delayed in infants with complex disabilities. This understanding is central for informing inclusive, generalizable theories of language development and strategies for supporting language development in infants with cerebral palsy.

The current study seeks to characterize the physical and attentional context of caregiver speech input during the period of early sitting development to test a central hypothesis suggested by past work: that being in a sitting position increases infants’ attention to objects during labeling. In particular, we aim to examine whether caregiver speech input during a free play interaction—frequency of utterances, frequency of object labels, and infant and caregiver attention during object labeling—differs when infants are sitting compared to other positions, and between typically developing infants and infants with cerebral palsy. We hypothesized that sitting would facilitate the provision of the “right label at the right time”; in other words, sitting would increase the probability that infant attention would co-occur with caregiver object labeling. Specifically, we predicted that infants would be more likely to be attending to labeled objects, would be more likely to be engaged in multimodal attention to labeled objects, and that infants and caregivers would be more likely to be engaged in coordinated visual attention to labeled objects, while infants were sitting compared to other postures. If this hypothesis were confirmed, it would suggest that sitting may increase the value of individual object labeling events. Because previous studies have demonstrated consistent impacts of postural skills and body position on social and object interaction regardless of whether those skills were achieved on a typical or delayed timeline [18,39,56], we did not hypothesize different patterns of results in infants with typical development and infants with cerebral palsy. In other words, we predicted that sitting’s impact on attention to labeled objects would be universal—that infants with typical development and infants with cerebral palsy would both be more likely to be attending and to coordinate attention to labeled objects while in a sitting position. Because children with cerebral palsy develop independent sitting later than those with typical development (if at all), and likely spend less time sitting during play, confirmation of this hypothesis would suggest that the facilitating effect of sitting may be delayed or missing in this population, which could impact language outcomes. Finally, in order to assess the role of maturation and development, we also examined the impact of infant age and sitting skill on speech input.

## Materials and methods

### Participants

A total of *N* = 50 infants (*n* = 22 infants with cerebral palsy, “CP”; *n* = 28 infants with typical development, “TD”) were included in this analysis. Infants were drawn from a larger sample of participants in a multisite clinical trial (CP group) and single-site companion study (TD group). Data at all sites were collected between March 9, 2016 and January 15, 2019; ethics approval at all data collection sites relied on a central IRB at the primary site. Characteristics of the sample are described in Table 1.

**Table 1:** Sample demographics.

	Typical Development (n = 28)	Cerebral Palsy (n = 22)
Child age (months)^a^	*M* = 5.68 (*SD* = 0.81)	*M* = 11.1 (*SD* = 3.08)
Child sex		
Female	12 (42.86%)	11 (50.00%)
Male	16 (57.14%)	11 (50.00%)
Child race		
White	21 (75.00%)	13 (59.09%)
Black	3 (10.71%)	5 (22.72%)
Asian	1 (3.57%)	1 (4.55%)
Multiple	3 (10.71%)	1 (4.55%)
Unknown or not reported	0 (0%)	2 (9.09%)
Child ethnicity		
Not Hispanic	27 (96.43%)	20 (90.09%)
Hispanic	1 (3.57%)	1 (4.55%)
Unknown or not reported	0 (0%)	1 (4.55%)
Primary caregiver highest education		
No high school diploma	1 (3.57%)	0 (0%)
High school diploma/GED	1 (3.57%)	4 (18.18%)
Some college	2 (7.14%)	2 (9.09%)
Bachelor’s degree	5 (17.86%)	8 (36.36%)
Post-graduate degree	19 (67.86%)	6 (27.27%)
Not reported	0 (0%)	2 (9.09%)
Household SES^b^		
Low to middle	4 (14.29%)	7 (31.81%)
High	24 (85.71%)	12 (54.54%)
Not reported	0 (0%)	3 (13.63%)
Child gestational age		
Full term (≥37 wk)	28 (100%)	8 (36.36%)
Moderate-late preterm (32–37 wk)	0 (0%)	4 (18.18%)
Very preterm (28–31 wk)	0 (0%)	4 (18.18%)
Extremely preterm (<28 wk)	0 (0%)	6 (27.27%)

^a^Represents adjusted age for infants born preterm (<37 weeks gestation).

^b^SES = socioeconomic status; low to middle = no college and/or poverty income ratio < 2, high = at least some college and poverty income ratio ≥ 2. *M* = mean, *SD* = standard deviation.

Infants with CP were recruited for the clinical trial from five sites across the United States. The purpose of the trial was to assess the efficacy of a physical therapy intervention focused on sitting, reaching, and motor based problem solving in infants with motor delay [57,58]. Therefore, infants began the trial at a consistent stage of sitting development: Infants were eligible to enroll if they were able to sit for at least 3s, using hands for support if necessary, but unable to transition in and out of sitting. Other eligibility criteria included age 7–16 months (corrected for prematurity if applicable), gross motor delay defined as a score lower than 1 SD below the mean on the gross motor subtest of the Bayley Scales of Infant Development, Third Edition [59], and ability to spontaneously move the arms in any position. Infants with medical, orthopedic, or visual disorders that would limit ability to participate in assessment and intervention focused on sitting and reaching (for example, blindness, recent surgery or movement precautions, or medical conditions that restricted upright positioning), infants with degenerative conditions, and infants with uncontrolled seizures were excluded. All infants’ parents/guardians provided written informed consent to participate in the study. For the current analysis, only infants with CP were selected for analysis. The subset included infants whose parents reported one of the following diagnoses, either at enrollment or one year post-enrollment: cerebral palsy, spastic cerebral palsy, hemiplegia, diplegia, quadriplegia, and/or stroke.

Infants with TD were recruited from a single site in the southeastern United States. They were enrolled at the same stage of sitting development as the CP group. Other eligibility criteria included age < 7 months and score higher than 1 SD below the mean on the Bayley gross motor subtest; infants were excluded if they had a history of preterm birth, developmental delay, or other medical complications. Note that, by definition, infants in the TD group were younger at enrollment than infants in the CP group.

Fourteen infants meeting the above criteria were excluded from the current analysis for the following reasons: the caregiver spoke a language other than English during the free play observation (4 CP, 2 TD), some caregiver speech was directed toward a sibling who was present during the free play observation (2 CP, 3 TD), two caregivers were present during the free play observation (1 TD), the infant cried throughout the free play observation (1 CP), or audio quality was too poor for transcription (1 TD).

### Free play observation procedure

The data examined in the current study are primarily from a free play observation conducted at the baseline assessment visit for the clinical trial or companion study. Visits were typically performed in participants’ homes but occasionally (6 infants with TD and 1 infant with CP) took place in a lab setting per parent preference. Preliminary examination of the data indicated outcomes were comparable between infants tested in the home vs. the lab. Several other standardized and play-based assessments were performed during the visit [57] but are not included in the present analysis.

Infants were recorded with a single video camera for 5 minutes of free play with a single caregiver. One infant in the CP group became fussy partway through, so only 3.3 minutes of free play were coded for that infant. Caregivers were instructed simply to play with their babies as they normally would. All caregivers were provided four standard toys (a ball, a car, a telephone-shaped rattle, and a linked chain) to ensure all dyads had access to manipulable and easily labeled objects, but participants could also play with any other objects in their environment. Caregivers were not instructed in how to position or talk to their infants or how to use the toys.

### Data coding

Free play observations were coded for the following behaviors using Datavyu software (datavyu.org).

#### Body position.

Coders scored moment by moment body position as either *sitting* (including independent sitting, sitting with assistance from a caregiver or on a caregiver’s lap, or sitting in a supportive device or seat), *non-sitting* (inclusive of time spent in prone, supine, or standing, and time being held off the floor by an adult) or *transitioning* (time moving between positions). (Note that 4 infants with CP and 2 infants with TD sat in an infant seat during part of the play observation; sitting in a seat comprised *M* = 7% of the total observation time in both groups. This was included as sitting as infants’ bodies were in a similar postural configuration to sitting on the floor or lap: trunk upright and base of support at the pelvis/thighs.) A primary coder scored 100% of videos, and a secondary coder scored a subset of 24% of the videos to assess inter-rater reliability. Coders agreed on *M* = 97.7% of video frames, Cohen’s κ = 0.955.

Note that due to the stage of motor development of the sample, non-sitting time primarily comprised lying on the floor in supine or prone positions. More detailed coding revealed that standing was very rare (*M* = 2% of the total observation time for both groups, typically only seconds at a time). Standing was included as non-sitting in analyses, as the focus of the current study was on the unique role of sitting in the early language learning environment. Notably, prior work has suggested that joint attention [16] and caregiver-provided cognitive learning opportunities [18] are elevated specifically in sitting and are similarly low in prone and standing positions. Regrouping the data to include standing time with sitting (creating an “upright” vs. “non-upright” dichotomy) does not change the results.

#### Caregiver utterances.

Caregiver utterances were transcribed in Datavyu using the definitions published for the Play and Learning Across a Year (PLAY) project (https://www.play-project.org/coding#Transcription, definitions for “mother utterances” only; [60]). An utterance was defined as “a unit of speech separated by grammatical closure, intonation contour, or prolonged pausing.”

Because videos were not originally collected with the intent to transcribe audio (e.g., microphones were not worn, cameras were not placed at a consistent distance from the caregivers, background noise was not controlled, etc.), ease of transcription was highly variable. Therefore, every video was separately transcribed by two independent coders and consensus was reached through discussion. Note that this analysis did not require knowing the precise timing of all utterances, but did require that the utterances be associated with the body position that the infant was in during the utterance. Therefore, coders entered transcriptions nested within the previously coded position windows, but did not code exact timestamps. As defined in the PLAY protocol, placeholders were entered for utterances that clearly occurred but were not clear enough to transcribe.

This coding provided the *number of total utterances* during the play observation. Interrater agreement on number of utterances was excellent, *ICC* = 0.978; coders agreed on the position window for *M* = 92% of utterances. All utterances, even ones that were not clear enough to transcribe, were counted in the number of utterances. As a more interpretable and generalizable number, we calculated the *utterance rate*, or the number of utterances per minute of play.

#### Object labels.

From the transcriptions, coders identified every time caregivers named an object. Objects included any concrete, tangible item that could potentially be looked at or touched. Nouns that did not count as objects included people’s names, pronouns (e.g., “you”, “it”, “that”), generic words (e.g., “thing”, “one”), patterns/textures (e.g., “stripes”, “bumps”), and places (e.g., “school”). We also excluded objects that were named in the context of a song (e.g., pat-a-cake), as these were frequently repeated a large number of times without referents. Finally, because the role of looking and touching in learning nouns that refer to non-manipulable objects is unclear, we excluded the following types of labels: types of people (e.g., “sister”), furniture (e.g., “seat”), clothing (e.g., “shirt”), and body parts (e.g., “tummy”). Two coders scored all utterances and agreed on the presence of object labels in *M *= 97.1% of utterances, Cohen’s κ = 0.902; all differences were resolved through discussion.

This coding provided the *number of object labels* uttered by the caregiver during the play observation, from which we calculated the *object label rate*, or the number of object labels uttered by the caregiver per minute of play.

Note again that a small number of caregiver utterances were inaudible, or not clear enough for the coders to transcribe. Overall, 4,336 utterances were scored: 4,327 (97.7% overall, range 86.3–100%) were transcribed and 99 (2.3% overall, range 0–13.7%) were inaudible. Of the transcribed utterances, 369 (8.5% overall, range 0–24.5%) contained object labels. Assuming the proportion of utterances that contained labels was the same for transcribed and inaudible utterances, we calculated the estimated number of labels that may have been missed for each infant; this number ranged from 0–1.6 (*M *= 0.15). Moreover, the proportion of utterances that were inaudible did not differ between the groups. Therefore, all utterances (transcribed and inaudible) were included when analyzing the total number of utterances, but only utterances with a valid transcribed object label were included in analyses of object labels.

#### Attentional states.

For each object label referring to a manipulable object, coders determined the precise timestamp at which the caregiver began to say the object name. Coders then viewed a 4s window around this time (2s before to 2s after) and determined whether the labeled object was present in the immediate play area. If the object was present, they scored the occurrence of four types of object attention from the video: *infant touch* (infant contacted the object with their hands at any point during the 4s window)*, infant look* (infant directed their gaze toward the object at any point during the window)*, caregiver touch* (caregiver contacted the object with their hands at any point during the window), and *caregiver look* (caregiver directed their gaze toward the object at any point during the window). If the object was occluded during the 4s window, coders viewed the video before and/or after the window to determine the location of the object. Because videos were collected in the home environment without a standardized camera setup, and because precise gaze tracking measurements were not collected in this study, there was some ambiguity in coding looking from a third person view. Therefore, coders were instructed to be liberal in their coding of looks; if the participant appeared to direct their gaze in the general direction of the object, this was scored as a look. All windows were scored by two coders and inter-rater agreement was *M* = 96.1% (κ = 0.918) for infant touch, *M* = 97.7% (κ = 0.886) for infant look, *M* = 98.3% (κ = 0.940) for caregiver touch, and *M* = 97.5% (κ = 0.574) for caregiver look; all disagreements were resolved through discussion. (Note that prior studies examining attention during object labeling have used both larger—e.g., 6s window around the labeling event [31] and smaller—e.g., simultaneous with labeling event [24]—durations for counting looking at and touching a labeled object. We selected 4s as an intermediate window; prior work shows that this window corresponds with peak holding and looking behavior [26].)

From these codes, we defined three attentional states that prior literature suggests are relevant for word learning: *infant attention* (infant touching and/or infant looking), *infant multimodal attention* (infant both touching and looking) [17], and *coordinated visual attention* (both infant and caregiver looking) [61]. These attentional states were not mutually exclusive; for example, if the infant was looking and touching and the caregiver was looking, this labeling event would be counted as infant attention, infant multimodal attention, *and* coordinated visual attention.

### Sitting assessment

In addition to the free play observation, infants were assessed using the Gross Motor Function Measure-88 (GMFM) Sitting dimension. The GMFM is a criterion-referenced measure of gross motor function designed and validated for children with cerebral palsy [62]. The Sitting dimension includes 20 items assessing sitting function including head control, sitting with and without arm support, and reaching while sitting. Each item is scored on a 4-point scale from 0 (does not initiate) to 3 (completes) for a total possible score of 60.

### Data analysis

All analyses were performed in R using the functions and packages listed with the descriptions below.

#### Handling of age and sitting skill.

To account for differences in chronological age, we ran all initial models including age as a covariate. As age was non-overlapping between the TD and CP groups, group*age interaction effects were also included. Age was always centered at the mean within each group so that coefficients represent effects for each group at the average age for that group. To simplify models, age was removed when not significant.

To assess effects of sitting skill, we repeated all models replacing age with GMFM sitting score as the covariate. GMFM sitting was centered at the mean so that coefficients represent effects for the average sitter. To simplify models, GMFM sitting was removed when not significant.

#### Sitting.

Because we were interested in the role of sitting behavior in caregiver speech input, we first describe the distribution of sitting behavior during the free play observation. Specifically, we examined the duration (as a proportion of the 5-minute play observation) infants spent in a sitting position. To assess whether sitting duration differed between the CP and TD groups and varied with sitting skill and age, we performed a linear regression (*lm* function) with sitting duration as the outcome variable and GMFM sitting score, age, group, and an age*group interaction as predictors.

#### Quantity of caregiver speech input—Utterances and object labels.

Number of caregiver utterances and number of object labels were measured as counts, or the number of occurrences in a defined period of time. (Figures show the rates of utterances and object labels per minute rather than raw counts to increase interpretability.) Because the distribution of values for number of utterances and number of object labels deviated considerably from a normal distribution, we considered both Poisson and negative binomial distributions to model each outcome. The negative binomial models were a better fit as indicated by smaller AIC and BIC values, so negative binomial models are reported here.

To examine how caregiver speech input differed based on infant position, we calculated the number of utterances and object labels *during sitting* and *during non-sitting*. Time transitioning between positions was removed from these analyses. Note that some infants sat for the entire observation and contributed data only to sitting, some never sat and contributed data only to non-sitting, and some sat for part of the observation and contributed data in both positions. We fit a generalized linear mixed model (*mixed_model* function from the *GLMMadaptive* package) with position (sitting vs. non-sitting), group (TD vs. CP), and position*group as fixed predictors and random intercepts for each participant. As described above, age and GMFM sitting (as well as age*group and GMFM*group) were initially included as predictors and removed when not significant. As is customary when modeling count variables (Poisson or negative binomial distributions), we included the total amount of time available for the events to occur as an offset variable [63]. Here, the offset was the total position duration (i.e., total duration sitting or non-sitting), to account for different amounts of time spent in each position between infants.

Note that the TD group and the CP group differed socioeconomically, with lower socioeconomic status (SES) reported in the CP group (Table 1). As child-directed speech is known to differ by SES [64], we explored SES as a predictor of caregiver utterances and object labels. As in prior work [65], a dichotomous measure of SES was calculated using the primary caregiver’s highest education level, household income, and number of individuals in the household. The poverty income ratio was calculated as household income divided by the poverty level for the household size. High SES was defined as having at least some college and a poverty income ratio ≥ 2 (i.e., at least twice the poverty level), and low to middle SES was defined as having no college and/or a poverty income ratio < 2 (less than twice the poverty level). SES was not a significant predictor of either variable, and including SES in the models did not change the effect of group. As SES was missing for three infants, it was not included as a predictor in the final models.

#### Attentional states during input.

Attentional states were measured dichotomously (occurred or did not occur) for each object labeling event. (Figures show the proportion of object labels that co-occurred with each attentional state.) To examine the probability of object labels co-occurring with attentional states, we fit generalized linear mixed models with a binomial distribution (*glmer* function from the *lmerTest* package). Note that the unit of analysis in these models is at the level of the object labeling event, not the infant. The models included position, group, and position*group as fixed predictors, random intercepts for participant, and an offset for the total position duration. Again, age and GMFM sitting and their interactions with group were initially included as predictors and removed when not significant.

#### Exploratory analyses: Sitting type and orientation.

Although our primary analyses collapsed all time that infants were in an upright sitting position into a single “sitting” category, prior work has documented differences in caregiver-infant interaction between independent and supported sitting [39]. Therefore, we were interested in exploring whether caregiver speech input differed between these two sitting types. In the current dataset, only 14 infants sat independently, and this was unbalanced between the groups (11 infants with TD and only 3 with CP). Additionally, the duration in independent sitting was relatively low (*M* = 10% of the total observation time for infants with TD and *M* = 5% for infants with CP). Therefore, statistical power for a rigorous comparison was limited. However, we explored this question by fitting new models for the primary outcome variables (negative binomial for number of object labels and binomial for attentional states), with only sitting type (independent vs. caregiver supported) as a predictor, collapsing over groups.

Relatedly, caregiver-infant interactions during sitting may be affected by the orientation of the infant relative to the caregiver. In particular, new and emerging sitters are often positioned in front of, and facing away from, a caregiver who provides manual support from behind [39]. In the current study, infants spent about half of their sitting time facing away from the caregiver (*M* = 59% of sitting time for infants with TD and *M *= 39% of sitting time for infants with CP). Potentially, this configuration could negatively impact communication and social attention during play. We therefore explored whether speech input and attentional states differed when infants were oriented “facing away” from the caregiver compared to when they were “facing toward” (including times when they were oriented directly face to face with the caregiver and times when they were oriented perpendicular to the caregiver) by fitting additional negative binomial and binomial models with infant orientation (facing away vs. facing toward) as the sole predictor.

Note that both exploratory analyses included only periods of time when infants were in a sitting position, from the 23 infants with TD and 17 infants with CP who sat during the play observation.

## Results

### Effects of age and sitting skill

None of the models revealed significant effects of age or age*group interactions. In other words, time sitting, number of utterances, number of object labels, and attentional states during labeling were unaffected by age within each group, with no differences in the effect of age between the groups. Similarly, none of the models revealed significant effects of GMFM sitting or age*GMFM interactions. Therefore, age and GMFM sitting were removed from the final models.

### Sitting

Infants varied in the amount of time spent sitting during the free play observation (or, more accurately, caregivers varied in the amount of time they placed their infants in a sitting position). Seven infants never sat and 11 infants sat for the full five minutes; for the other 33 infants, sitting ranged from 12–99% of the free play observation. The proportion of time sitting was not associated with age (*B* = 0.012, *SE* = 0.056, *p* = 0.835) or sitting skill (*B* = -0.007, *SE* = 0.013, *p* = 0.603) and did not differ between CP and TD groups (*B* = -.080, *SE* = 0.122, *p* = 0.513).

### Quantity of speech input

**Fig 1:**
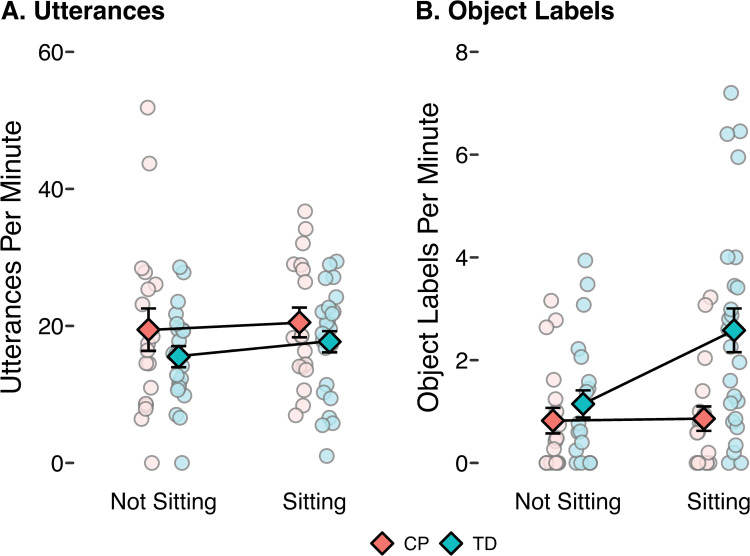
Quantity of caregiver speech input during non-sitting and during sitting. **A)** Rate of caregiver utterances. **B)** Rate of object labels. Light colored circles depict values for individual infants and darker colored diamonds depict the group means. Error bars represent standard error of the mean. CP = infants with cerebral palsy, TD = infants with typical development.

The number of utterances also varied widely between caregivers, from a minimum of 1 per minute to a maximum of 38.2 per minute (*M* = 17.4 per minute; Fig 1). Number of utterances did not differ by group or during sitting compared to non-sitting (Table 2). Note that the direction and significance of these effects are unchanged when two outliers (infants with CP who heard 51.9 and 43.7 utterances per minute while in a non-sitting position) are removed.

**Table 2:** Model results: Utterances and object labels.

	Utterances			Object Labels		
PRIMARY ANALYSES						
	Estimate	SE	p	Estimate	SE	p
Intercept	-8.237	0.076	<.001	-10.792	0.146	<.001
Position	0.118	0.071	0.098	**0.445**	**0.210**	**0.034**
Group	0.164	0.151	0.275	**-0.636**	**0.241**	**0.008**
Position*Group	0.060	0.144	0.679	-0.675	0.440	0.125
FOLLOW-UP ANALYSES						
*Effect of infant object contact*						
Intercept				-10.790	0.145	<.001
Position				**0.444**	**0.210**	**0.034**
Group				**-0.654**	**0.258**	**0.011**
Position*Group				-0.686	0.442	0.121
Object Contact				-0.087	0.446	0.846
*Effect of caregiver object contact*						
Intercept				-10.792	0.146	<.001
Position				**0.451**	**0.226**	**0.046**
Group				**-0.638**	**0.242**	**0.008**
Position*Group				-0.671	0.448	0.134
Object Contact				-0.038	0.550	0.945

Estimates are on the log scale. All models include random intercepts for participant. Group was dummy coded with TD = 0 and CP = 1, and then centered at the mean. Position was dummy coded with not sitting = 0 and sitting = 1, and then centered at the mean. Object Contact (representing the proportion of sitting/non-sitting time that the infant or caregiver was contacting an object) was centered at the mean. Bold numbers represent statistically significant effects (α=0.05). SE = standard error of the estimate.

Most utterances served functions other than labeling objects. Caregivers used speech to direct or describe infants’ actions (e.g., “Let’s sit back up” or “You just wanna chew.”), to provide affirmations (“Good job!” or “Yay!”), and to obtain infants’ attention (“Look over here!”). Many utterances referred to objects without using the object’s name (e.g., “You love those!” or “Can you catch it?”). However, the majority of infants (48/50) were exposed to some object labels. On average, caregivers labeled 1.5 objects per minute (range = 0–7.2 per minute; Fig 1). Caregivers mostly labeled the provided objects, although 62/369 labels (17%) referred to other objects.

Object labeling varied by group and body position: Infants with TD were exposed to more labels than infants with CP, and infants were exposed to more labels while in a sitting position than while in other positions (Table 2). In fact, frequency of object labeling was nearly double while infants were sitting (*M* = 1.0 labels per minute non-sitting and *M* = 1.9 labels per minute sitting).

Note that four infants with TD had especially high values for object labels (over five labels per minute). These data points were not statistical outliers as the values were within 1.5 times the interquartile range (see S1 File). However, we examined these infants’ data closely to determine whether they were unique in any other ways. We found no indicators that these infants or caregivers were unusual or that there were any errors in the procedure: The dyads were not outside the typical range in any other measured variables, all interacted mainly with the provided toys, they were split evenly between sitting independently (two infants) and sitting only with support (two infants), and they all spent some time in both orientations (facing away and facing toward).

### Attentional states during input

Labeled objects were almost always present in the play area (*M* = 95% of labels, range = 50%-100% between infants). Overall, looking was more common than touching and caregiver attention was more common than infant attention. Infants looked at the object on *M* = 75% of labeling events (range = 0–100%) and touched the object on *M* = 39% of labeling events (range = 0–100%); caregivers looked at the object on *M* = 91% of labeling events (range = 50–100%) and touched the object on *M* = 74% of labeling events (range = 0–100%). Infant attention (*M* = 80% of labels, range 0–100%) and coordinated visual attention (*M* = 72% of labels, range 0–100%) occurred frequently, but infant multimodal attention was infrequent (*M* = 34% of labels, range 0–100%). Only *M* = 20% (range = 0–100%) of labeling events contained no attentional cues from either member of the dyad.

**Fig 2:**
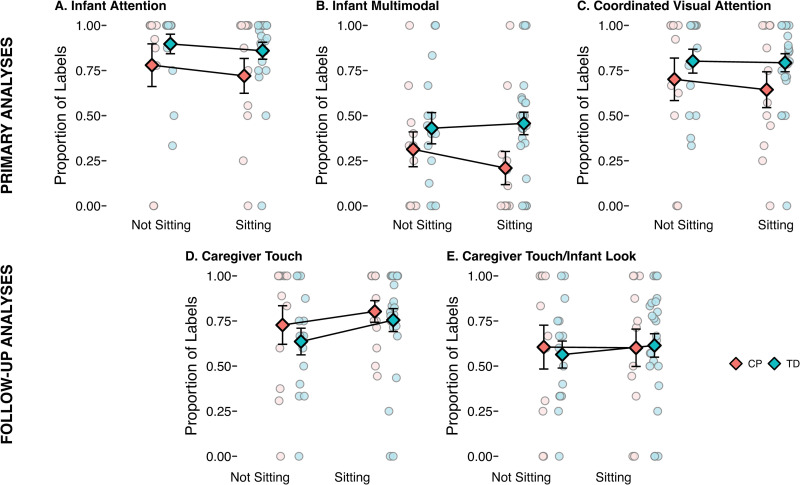
Proportion of object labels with each attentional state during non-sitting and during sitting. **(A)** Proportion of labels that co-occurred with any infant attention (looking *or* touching). **(B)** Proportion of labels that co-occurred with infant multimodal attention (looking *and* touching). **(C)** Proportion of labels that co-occurred with coordinated visual attention (both caregiver and infant looking). **(D)** Proportion of labels that co-occurred with caregiver touching. Light colored circles depict values for individual infants and darker colored diamonds depict the group means. Error bars represent standard error of the mean. CP = infants with cerebral palsy, TD = infants with typical development.

We hypothesized that while infants were in a sitting position, they would be more likely to be attending to (looking *or* touching) labeled objects, they would be more likely to be engaged in multimodal attention (both looking *and* touching), and they would be more likely to coordinate attention (infant *and* caregiver looking). However, there was no difference in the probability of these attentional states between sitting and non-sitting positions (Fig 2, Table 3). There was a significant effect of group for infant attention and infant multimodal attention; infants with TD were more likely than infants with CP to be attending to objects, multimodally and overall, when they were labeled (Table 3).

**Table 3:** Model results: Attentional states.

PRIMARY ANALYSES									
	Infant Attention			Infant Multimodal Attention			Coordinated Visual Attention		
	Est	SE	p	Est	SE	p	Est	SE	p
Intercept	1.970	0.166	<.001	-0.517	0.148	<.001	1.287	0.148	<.001
Position	-0.208	0.365	0.569	0.007	0.274	0.981	-0.053	0.294	0.858
Group	**-0.874**	**0.328**	**0.008**	**-0.824**	**0.334**	**0.014**	-0.507	0.304	0.096
Position*Group	-0.839	0.696	0.228	-1.162	0.600	0.053	-0.835	0.600	0.164
FOLLOW-UP ANALYSES									
	Caregiver Touch			Caregiver Touch/ Infant Look					
	Est	SE	p	Est	SE	p			
Intercept	1.044	0.190	<.001	0.474	0.158	0.003			
Position	0.323	0.310	0.297	0.057	0.290	0.844			
Group	0.076	0.388	0.845	-0.035	0.335	0.918			
Position*Group	0.048	0.651	0.941	0.116	0.590	0.845			

Estimates are on the logit scale. All models include random intercepts for participant. Group was dummy coded with TD = 0 and CP = 1, and then centered at the mean. Position was dummy coded with not sitting = 0 and sitting = 1, and then centered at the mean. Bold numbers represent statistically significant effects (α = 0.05). SE = standard error of the estimate.

### Independent vs. supported sitting

The exploratory analysis suggested that object labels were considerably more frequent in independent compared to supported sitting (*M* = 4.0 vs. *M* = 1.7 labels per minute; *B* = 0.575, *SE* = 0.248, *p* = 0.020), and that infant multimodal attention was more frequent in independent compared to supported sitting (*M* = 63% vs. *M* = 32% of labels; *B* = 0.868, *SE* = 0.369, *p* = 0.019). The effect of sitting type was not significant for infant attention (*B* = -0.107, *SE* = 0.533, *p* = 0.842) or coordinated visual attention (*B* = 0.099, *SE* = 0.419, *p* = 0.812).

### Infant-caregiver orientation

The second exploratory analysis found no evidence that facing away from the caregiver while sitting negatively impacted caregiver speech input. The effect of orientation was not significant for number of object labels (*B* = 0.1801, *SE* = 0.1839, *p* = 0.328), infant attention (*B* = 0.9607, *SE* = 0.5385, *p* = 0.0744), or infant multimodal attention (*B* = 0.5189, *SE* = 0.2969, *p* = 0.0805); coordinated visual attention was significantly *more* frequent while infants faced away than while they faced toward the caregiver (*M* = 87% vs. *M* = 63% of labels; *B* = 0.9540, *SE* = 0.3859, *p* = 0.0134).

### Follow-up analyses

The results were unexpected: Attention to labeled objects (infant attention, infant multimodal attention, and coordinated visual attention) was not more likely while sitting than while not sitting, but caregivers labeled objects more while infants were sitting. We therefore performed additional analyses to examine new hypotheses generated by these findings.

#### Role of caregiver object manipulation during labeling events.

We initially hypothesized that infants would have improved visual and manual access to objects in a sitting position, which would lead to a higher likelihood of attending to objects at the time of labeling. An upright sitting position leads to a wider view of the surroundings [40], a better view of caregivers’ faces and increased joint attention [16], and increased coordinated multimodal object exploration [17]. Why, then, were infants equally likely to attend to labeled objects while in prone or supine positions which, presumably, make visual and multimodal attention to objects more challenging? One possibility is that caregivers compensate for this challenge by actively manipulating objects and bringing them into infants’ view during labeling while in non-sitting positions. Especially early in development, object interaction is scaffolded by caregivers’ holding and manipulating objects, making them salient to infants and potentially providing cues that could be helpful for word learning [29,66,67]. We tested this by examining two additional measures not included in our initial planned analyses: 1) the probability of caregivers touching the labeled objects, termed *caregiver touch*, and 2) the probability of caregivers touching the labeled objects while infants also looked at the labeled objects, termed *caregiver touch/infant look*. We fit two generalized linear mixed models using the strategy described above for the other attentional states, with caregiver touch and caregiver touch/infant look as the binary outcomes and position, group, and position*group as fixed predictors. For caregiver touch, contrary to the hypothesis, caregivers were slightly *less* likely to touch labeled objects when infants were not sitting (Fig 2d) than when they were sitting. The models revealed no significant effects of position, group, or their interaction for either variable (Table 3).

#### Object manipulation during sitting and non-sitting.

What might account for the increased rate of object labeling during sitting? Although sitting was not associated with the infants’ and caregivers’ likelihood of touching labeled objects *during labeling events*, it is possible that *overall rates* of object manipulation play a role in boosting the frequency of object labels. Previous work suggests that caregivers are more likely to provide labels when their infants are holding or touching objects [24,25,68], and that infants spend more time holding objects while in a sitting position [69]. Therefore, it is possible that increased time manipulating objects in sitting prompts caregivers to label more objects.

We tested this possibility by incorporating coding of infant and caregiver object contact throughout the full play observation. Object contact was coded any time the infant or caregiver’s hand was in contact with a manipulable object, excluding furniture, surfaces, or body parts [39]. A subset 12% of videos for infant object contact and 14% of videos for caregiver object contact were double-coded to assess interrater reliability; coders agreed on *M* = 98.4% of video frames for infant object contact, Cohen’s κ = 0.967, and *M* = 96.9% of video frames for caregiver object contact, Cohen’s κ = 0.938. For each participant, we calculated the total proportion of sitting time contacting objects (time contacting objects and sitting/ total time sitting) and the total proportion of non-sitting time contacting objects (time contacting objects and not sitting/ total time not sitting). To determine whether the proportion of time contacting objects differed by position, we fit linear mixed models with proportion of time contacting objects as the outcome; position, group, and position*group as fixed predictors; and a random intercept for participant. Then, to determine whether the proportion of time contacting objects influenced object labeling, we added the proportion of time contacting objects as a covariate to our generalized linear mixed models predicting the number of object labels (position, group, position*group, and object contact as fixed predictors, random intercept for participant, and offset of total position duration).

**Fig 3:**
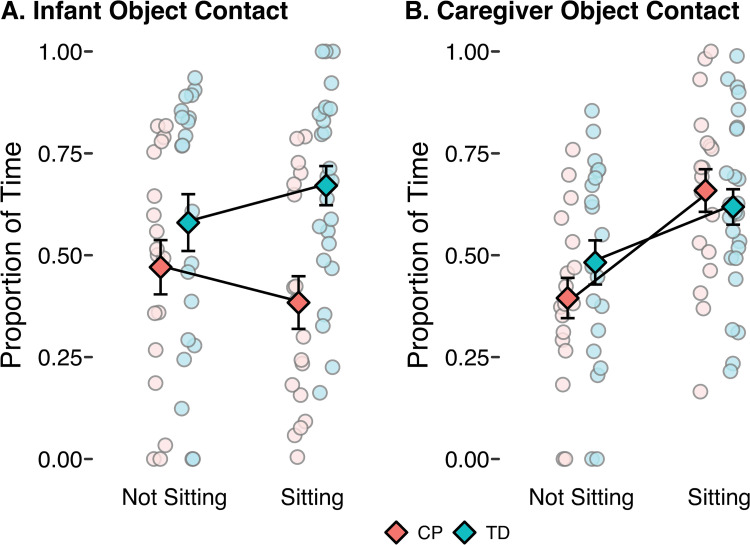
Proportion of non-sitting time and sitting time with object contact. **A)** Proportion of time with the infant contacting an object. **B)** Proportion of time with the caregiver contacting an object. Light colored circles depict values for individual infants and darker colored diamonds depict the group means. Error bars represent standard error of the mean. CP = infants with cerebral palsy, TD = infants with typical development.

***Infant object contact:*** Overall, infants spent 54% of the play observation contacting objects (43% for infants with CP and 63% for infants with TD). The first model revealed that infants with TD spent significantly more time contacting objects than infants with CP, but there was no difference in infant object contact between sitting and non-sitting (Fig 3, Table 4). The second model revealed that infant object contact time was not a significant predictor of object labeling, and that the reported effects of position and group remained when infant object contact was included in the model (Table 2).

**Table 4:** Model results: Proportion of time contacting objects.

	Infant Object Contact			Caregiver Object Contact		
	Estimate	SE	p	Estimate	SE	p
Intercept	0.537	0.035	<.001	0.546	0.025	<.001
Position	0.020	0.048	0.686	**0.192**	**0.049**	**<.001**
Group	**-0.196**	**0.071**	**0.008**	-0.022	0.051	0.667
Position*Group	-0.187	0.097	0.060	0.129	0.098	0.195

All models include random intercepts for participant. Group was dummy coded with TD = 0 and CP = 1, and then centered at the mean. Position was dummy coded with not sitting = 0 and sitting = 1, and then centered at the mean. Bold numbers represent statistically significant effects (α = 0.05). SE = standard error of the estimate.

***Caregiver object contact:*** Overall, caregivers also spent 54% of the play observation contacting objects (53% for caregivers of infants with CP and 56% for caregivers of infants with TD). The first model revealed that caregivers spent significantly more time contacting objects while infants were sitting (Fig 3, Table 4). However, the second model revealed that caregiver object contact time was not a significant predictor of object labeling, and that the reported effects of position and group remained when caregiver object contact was included in the model (Table 2).

## Discussion

Consistent with our overall hypothesis, we found that caregiver speech input to young infants with TD and infants with CP was affected by sitting. However, the pattern of results was unexpected. Contrary to our predictions, the attentional context of object labeling did not differ with body position. When caregivers labeled objects, infants were equally likely to be attending, to be attending multimodally, or to be engaged in coordinated visual attention to the object when they were sitting compared to when they were not sitting. However, we found that infants were exposed to nearly twice as many object labels per minute while in a sitting position. Speech input also differed between infants with TD and infants with CP: Caregivers of infants with TD produced more object labels, and infants with TD were more likely to be attending, and attending multimodally, to the object being labeled.

Why do caregivers label objects more often while their infants are sitting? One hypothesis, examined in follow-up analysis, was that caregivers respond to infant object manipulation by labeling the objects, and infants are more likely to manipulate objects while they are sitting. This hypothesis has two components: 1) infants touch objects more while sitting, and 2) the amount of time touching objects is associated with caregiver object labeling. Neither of these turned out to be true. Contrary to previous work [69], we found that infants were not more likely to contact objects while they were sitting than while they were in other positions. (Note that the cited study included data from older, mobile infants in a wide range of daily activities. In that population, object holding may be increased during activities commonly performed in a sitting position, such as stationary play or feeding, and depressed during activities commonly performed in other positions, such as locomotion. Our findings suggest that in the early stages of sitting development, within the context of dyadic play, infants touch objects at similar rates while sitting and non-sitting.) Moreover, the amount of time infants contacted objects was unrelated to the number of object labels their caregivers produced, and the effect of position remained significant when accounting for infant object contact. Therefore, our finding of increased object labeling while sitting does not seem to be due to increased object manipulation.

We also examined relations between sitting, object labeling, and caregiver object interaction. Unlike infant object contact, caregiver object contact was considerably more frequent while infants were sitting. Possibly, this reflects caregivers showing objects to infants when they were in a better position to see them, or engaging in more interactive object play such as rolling a ball or stacking blocks. This finding aligns with previous work showing increased caregiver-provided cognitive learning opportunities when infants were in a sitting position [18]. Although interesting, this pattern also did not explain the relation between infant sitting and caregiver object labeling; caregiver object contact was not associated with object labeling, and infant position remained a significant predictor of object labeling when caregiver object contact was included in the model.

What, then, accounts for the increased rate of object labeling during sitting? One possibility is that the difference is due to improved visual access to the environment in a sitting position. Compared to prone or supine, where the trunk is horizontal and the head points at the floor or the ceiling, sitting provides a vertical trunk position and an effortless panoramic view of the surroundings [40]. Therefore, infants are better able to visually attend to objects, and to engage in joint attention with caregivers, from a sitting position [16,70]. Caregivers may be sensitive to this shift in visual perspective, and respond to infants’ increased capacity for social and object attention by labeling objects [68,71]. It is notable that 75% of object labeling events co-occurred with the infant looking toward the labeled object, suggesting that caregivers may deliberately draw infants’ attention toward a labeled object and/or use infant gaze as a cue to label an object. (Note that any visual advantage of the upright sitting position is not likely to be tied to an increase in face-to-face interactions with caregivers. Prior work has shown that face-to-face interactions are actually most common in a supine lying position and decrease with age [41,72,73]. Moreover, exploratory analyses in the current study found that the rate of object labeling did not change when infants faced away from their caregivers. Rather, it is likely increased visual access to objects in the environment during sitting that cues an increase in object labeling.)

Testing this hypothesis would require measuring infant visual attention toward objects and caregivers throughout the full play session. Unfortunately, unlike object touching, moment-to-moment visual attention in a complex, unconstrained environment cannot be reliably coded from a third person video. Continuous measurements of visual attention require a simplified experimental setup (for example, two displays side-by-side placed in front of the infant) or an eye tracker to specifically record gaze direction. Ongoing research using head-mounted eye tracking during early dyadic play is currently underway to test this important missing link.

Other factors not measured in the current study may have also contributed to the increased rate of object labeling during sitting. For example, the upright position of the ribs and diaphragm during sitting has been theorized to facilitate support of the vocal apparatus and result in longer, more mature vocalizations [74]; other work has demonstrated that amount and properties of caregiver speech vary with the quality of infant babbling [75–77]. Associations between sitting, babbling, and object labels should be examined in future work to provide further insight into the contingent interactions that may support language learning. Moreover, although prior work has linked sitting to quality of object manipulation [17] and quality of object manipulation to object labeling [24], it would be informative to investigate how these processes interact in real time.

This cross-sectional study examined the real-time effects of being in a sitting position on infant language learning interactions. What implications might these findings have for developmental processes? Although not tested here, the increase in object labeling while infants are sitting suggests one potential mechanism for the link between sitting development and language development. Rather than, as predicted, increasing the *value* of object labeling by increasing the likelihood that a label will correspond to the focus of infants’ attention, sitting seems to increase the *quantity* of object labeling by cueing caregivers to label objects more often. Potentially, independent sitting development leads to more time in a sitting position in which to experience a high rate of object labels. If, as our data suggest, a majority of these occur during coordinated attention and over a third occur with the infant attending multimodally—the “right label at the right time”—infant sitting could increase the number of optimal language learning moments and improve early word learning. Moreover, our exploratory analysis suggested that object labels may be particularly frequent while infants are sitting independently compared to sitting with support. (Caution is required when interpreting this result as independent sitting was relatively rare in this sample and confounded with group.) This hypothesis is worth testing longitudinally with a large sample, longer naturalistic observation periods, and comprehensive language outcome measures to characterize an important developmental cascade initiated early in infancy.

The lack of significant position*group interaction effects suggests that the impact of sitting on caregiver speech input may be similar in younger infants with TD and older infants with CP at the same stage of sitting development. However, the data show a tendency for decreased language learning opportunities overall—fewer object labels and a lower proportion of object labels with infant attention—in infants with CP. These findings are consistent with previous work in infants born very preterm documenting decreased contingency between maternal speech and infant attention [78]. Although eligibility for the clinical trial providing the data for the current study was based on gross motor delay and delayed sitting development, many infants with CP have co-occurring impairments in oculomotor or fine motor skills. Therefore, they may be less likely to provide the attentional cues that elicit object labels and less likely to be attending when object labels do occur. Indeed, we found that infants with CP spent less of the play observation contacting objects than infants with TD. Additionally, caregivers of infants with CP may decrease their object labeling to match their perception of the child’s developmental level or receptive language abilities, to focus on challenging motor skills during play, or in response to fewer vocalizations or social bids by the child. Again, we note that group was confounded with sitting type; if independent sitting truly boosts object labeling, then the group effects may be due to the fact that only three infants with CP sat independently during the play session. It is important to note that CP is an umbrella term encompassing a wide variety of topographical distributions (unilateral vs. bilateral, upper vs. lower extremities) and levels of gross motor function. We did not have detailed information on type and severity of CP in the current sample, but it is likely that these factors influenced caregiver interaction and infant attention during play. Future studies should examine factors influencing caregiver speech input to children with CP to determine promising avenues for early intervention.

The focus on speech input during the time of early sitting development and the inclusion of infants with typical and atypical sitting development are important novel contributions of the current study. A substantial limitation of this study is the short observation window in which infants and caregivers were recorded. Five minutes of focused play may not be representative of everyday dyadic interactions and the complex ways in which body position and speech input intersect throughout a variety of contexts and daily routines. Moreover, caregivers were aware that they were being video recorded, which may have changed the rate or pattern of utterances and object labels. Additionally, infants’ and caregivers’ looking behavior was scored liberally from a third person view, and some labeling episodes may have been coded as containing looks to the target object when participants were truly looking at something else. The use of extended naturalistic observations [19], remote measurement methods [79–81], and eye tracking or first person video [31,82,83] will deepen our understanding of how everyday sensorimotor experiences and social interactions influence development.

## Supporting information

S1 File Alternative analysis of object labels. (DOCX)
