# Supplementary material for: Sitting and caregiver speech input in typically developing infants and infants with cerebral palsy
Source: PLoS One. 2025 May 23;20(5):e0324106. doi: 10.1371/journal.pone.0324106 (PMC12101734; doi:10.1371/journal.pone.0324106)
Supplement: S1 File — (DOCX) [file pone.0324106.s001.docx]

## Alternative analysis of object labels

Four infants with TD experienced >5 labels per minute while sitting. We re-ran our analysis of object labels, noting that the decrease in sample size and effect size would necessarily decrease power to find a statistically significant effect. We found that despite losing statistical significance, the pattern of results remained the same. In the full data set, labels were *1.86 times more frequent while sitting* (1.00 vs. 1.86 per minute); in the data set with the four infants removed, labels were still *1.60 times more frequent while sitting* (0.87 vs. 1.39 per minute). Therefore, we conclude that the result is not solely driven by these four infants.

**Supporting Figure: Rate of object labels during non-sitting and during sitting, with four infants with high rates of object labeling removed.** Light colored circles depict values for individual infants and darker colored diamonds depict the group means. Error bars represent standard error of the mean. CP = infants with cerebral palsy, TD = infants with typical development.

**Supporting Table: Model results for object labels, with four infants with high rates of object labeling removed.** Estimates are on the log scale. Model includes a random intercepts for participant. Group was dummy coded with TD = 0 and CP = 1, and then centered at the mean. Position was dummy coded with not sitting = 0 and sitting = 1, and then centered at the mean. SE = standard error of the estimate.

|  |  | Estimate | SE | p |  |
| --- | --- | --- | --- | --- | --- |
|  | Intercept | -10.885 | 0.131 | <.001 |  |
|  | Position | 0.297 | 0.217 | 0.171 |  |
|  | Group | -0.403 | 0.223 | 0.071 |  |
|  | Position*Group | -0.567 | 0.437 | 0.195 |  |
